# Identification of Potential Plasma Biomarkers for Abdominal Aortic Aneurysm Using Tandem Mass Tag Quantitative Proteomics

**DOI:** 10.3390/proteomes6040043

**Published:** 2018-10-18

**Authors:** Anders E. Henriksson, Markus Lindqvist, Carina Sihlbom, Jörgen Bergström, Dan Bylund

**Affiliations:** 1 Department of Laboratory Medicine, Sundsvall County Hospital, SE-851 86 Sundsvall, Sweden; markus.lindqvist@rvn.se; 2 Department of Surgery, Sundsvall County Hospital, SE-851 86 Sundsvall, Sweden; 3 Department of Natural Sciences, Mid Sweden University, SE-851 70 Sundsvall, Sweden; dan.bylund@miun.se; 4 Proteomics Core Facility, University of Gothenburg, SE-405 30 Gothenburg, Sweden; carina.sihlbom@gu.se (C.S.); jorgen.bergstrom@gu.se (J.B.)

**Keywords:** aortic aneurysm, biomarker, bleomycin hydrolase, clinical proteomics, mass spectrometry, proteomics

## Abstract

Plasma biomarkers that identify abdominal aortic aneurysm (AAA) rupture risk would greatly assist in stratifying patients with small aneurysms. Identification of such biomarkers has hitherto been unsuccessful over a range of studies using different methods. The present study used an alternative proteomic approach to find new, potential plasma AAA biomarker candidates. Pre-fractionated plasma samples from twelve patients with AAA and eight matched controls without aneurysm were analyzed by mass spectrometry applying a tandem mass tag (TMT) technique. Eight proteins were differentially regulated in patients compared to controls, including decreased levels of the enzyme bleomycin hydrolase. The down-regulation of this enzyme was confirmed in an extended validation study using an enzyme-linked immunosorbent assay (ELISA). The TMT-based proteomic approach thus identified novel potential plasma biomarkers for AAA.

## 1. Introduction

The pathophysiology of the development, progression and rupture of abdominal aortic aneurysm (AAA) is still not completely described [1,2]. There are currently no non-surgical prevention and management options leaving the patients with a pre-symptomatic elective repair as the sole option to prevent rupture [1]. AAA screening programs have been introduced in an attempt to reduce mortality due to rupture in the general population. However, most clinicians agree that only an AAA diameter above 5.0–5.5 cm generally justifies elective repair, and that small AAAs are best managed by ultrasonographic surveillance due to a very low rupture rate.

Human plasma is one of the most important proteomes from a clinical and medical point of view [3]. Recent reviews have expressed the need for a blood plasma biomarker to identify aortic rupture risk and thereby assist in stratifying patients with small screen detected aneurysms [4,5,6]. Identification of such circulating biomarkers with traditional hypothesis driven studies has hitherto been unsuccessful. More recently, non-hypothesis-driven mass spectrometry (MS) based proteomic approaches have been used to discover new clinically useful biomarkers for AAA [7,8,9,10,11,12]. These MS-based studies employed different study designs according to population, sampling, sample preparation, protein separation and identification, without establishing a preferential method for biomarker identification [13]. The present study aimed to identify new biomarker candidates for AAA management. For this purpose, MS analysis of depleted and Tandem Mass Tag (TMT) labelled plasma samples were used to discover differences in the protein profile between patients with AAA and controls without aneurysm.

## 2. Methods

### 2.1. Subject Sample

The study was performed in accordance with the principles of the Declaration of Helsinki. A total of 174 patients and control subjects participated in the investigation after signing an informed consent form approved by the regional ethics committee. The patients and controls were of same ethnic origin, and had similar socio-economic backgrounds, but were stratified by sex, age, and smoking habit. None of the participants had co-existing malignant disease, uremia, diabetes, and neither statin nor anticoagulant therapy. Twenty participants were selected for an initial discovery study, while all participants were included in a subsequent validation study. For the discovery study, twelve male AAA patients and eight male controls without AAAs were carefully selected to minimize possible bias [13,14]. Both patients and controls were former smokers. The average age (range) was 71 (59–83) years in the AAA group and 70 (62–78) years in the control group.

The 174 participants in the validation study were selected to give similar age, sex and current smoking habits in the controls and AAA patients (Table 1). The validation study also included ten patients that were reinvestigated three years after they had undergone surgical treatment for non-ruptured AAA. These follow-up patients were treated by conventional open AAA repair through a standard midline laparotomy incision and transperitoneal approach with infrarenal polyester graft repair.

Both AAA patients and controls underwent ultrasonography investigation of the abdominal aorta. Peripheral venous blood samples were then taken from controls and patients. Samples were centrifuged within 30 min at 2000 *g* for 20 min and aliquots of citrated plasma were frozen and stored at −70 °C until analysis.

### 2.2. Discovery Study

The workflow of the discovery study included the following sequential steps as described in detail below: depletion, tryptic digestion, TMT labelling and sample pooling, fractionation, desalting, analysis by liquid chromatography-tandem mass spectrometry (LC-MS/MS), database search, TMT quantification, and statistical analysis.

#### 2.2.1. Sample Preparation

Samples were depleted of high abundance plasma proteins (albumin and IgG) using the ProteaPrep Albumin and IgG Depletion kit (Protea Biosciences, Inc., Morgantown, WV, USA) according to the manufacturer’s instructions. For relative quantification (performed using 6-plex TMT-labeling, Pierce Biotechnology, Inc., Rockford, IL, USA), 10 µL aliquots from 20 randomly selected samples were pooled to create a standard protein mix. This standard pool was depleted in the same way as the samples and included as a reference in each prepared TMT-set (details on the TMT-sets are provided in Supplementary Table S1).

The elution from the depletion step was evaporated to dryness (Savant SpeedVac™, Thermo Scientific, Basel, Switzerland) before proceeding with the Filter-Aided Sample Preparation (FASP) protocol for tryptic digestion [15]. In brief, samples were solubilized in 50 µL of 4% SDS, 250 mM triehtylammonium bicarbonate (TEAB, Fluka, Sigma Aldrich, St. Louis, MO, USA) with 0.1 mM DTT. Protein concentrations were measured using the Pierce 660 nm protein kit (Thermo Scientific, Basel, Switzerland) according to the manufacturer’s guidelines. For each sample, 100 µg of total protein was digested using a 1:20 trypsin:protein ratio in a two-step digestion approach (sequencing grade modified porcine trypsin, Promega, Madison, WI, USA).

TMT labelling was performed according to the manufacturer’s recommendations with minor modifications. Specifically, reactions were quenched after 1 h by the addition of hydroxylamine to a final concentration of 0.25%. Labeled samples were combined into one set of samples prior to strong cation exchange (SCX) fractionation (100 × 2.1 mm PolySULFOETHYL A column, 5 µm particles, 300 Å pore size, PolyLc Inc., Columbia, MD, USA) using an ÄKTA purifier system (GE Healthcare Life Sciences, Uppsala, Sweden). At a flow rate of 0.25 mL/min the following gradient was applied: 100% A (25 mM ammonium formate, pH 2.8 in 25% acetonitrile) for 10 min; 0–20% B (500 mM ammonium formate, pH 2.8 in 25% acetonitrile) for 20 min; 20–40% B for 10 min and 40–100% B for 10 min and 100% B held for 10 min. UV absorbance at 280 nm was monitored while fractions were collected in tubes at 0.5 mL intervals. Per TMT-set, 18 fractions were collected for analysis by nano LC-MS/MS. Prior to this analysis, the SCX-fractions were desalted using Pierce C18 spin columns (Pierce Biotechnology, Inc., Rockford, IL, USA).

#### 2.2.2. Nano LC-MS Analysis

Samples were analyzed on a Q Exactive mass spectrometer interfaced with an Easy-nLC 1000 nano LC system (both Thermo Fisher Scientific, Inc., Waltham, MA, USA). Peptides (3 µL injection volume) were separated using an in-house constructed pre-column and analytical column set up (45 mm × 0.075 mm I.D. and 200 mm × 0.750 mm I.D., respectively) packed with 3 μm Reprosil-Pur C18-AQ particles (Dr. Maisch GmbH, Ammerbuch, Germany). The following gradient was run at 150 nL/min; 7–27% B-solvent (acetonitrile in 0.2% formic acid) over 60 min, 27–40% B over 10 min, 40–80% B over 5 min with a final hold at 80% B for 10 min.

The mass spectrometer was operated in a so-called Top10 data acquisition approach. Full scan MS spectra were taken at a resolution of 35,000 (full width at half maximum, FWHM, at 200) over an *m/z* range of 400–1800 with an automatic gain control (AGC) target value of 3 × 10^6^ and a maximal injection time of 100 ms. An ion from ambient air at *m/z* 445.12003 was used for internal calibration.

Based on each full scan spectrum, the 10 most abundant ions above an intensity of 1.0 × 10^5^ (“underfill ratio”: 0.7%) were selected for fragmentation using a stepped higher energy collisional dissociation regime (stepped HCD, NCE 35 +/− 25%) and an isolation width of 2 Da. Unassigned, singly charged as well as ≥5 charge states were excluded from fragmentation and fragmented precursor masses were excluded dynamically for 30 s. Fragment spectra were recorded at a resolution of 17,500 for a target AGC-value of 1 × 10^6^ and a maximal injection time of 64 ms was used for MS/MS scans.

For increased coverage and sensitivity using an exclusion list strategy, samples were injected a second time using a longer gradient and with exclusion lists based on the identifications made in the first round of analysis. The following gradient was run at 150 nL/min: 7–25% B-solvent (acetonitrile in 0.2% formic acid) over 90 min, 25–40% B over 10 min, 40–80% B over 10 min with a final hold at 80% B for 15 min. A smaller mass range (*m/z* 300–1400) was used compared to the first analysis. For the exclusion list runs, the 10 most abundant ions above an intensity of 1.0 × 10^4^ (“underfill ratio”: 0.1%) were selected for fragmentation using a stepped higher energy collisional dissociation regime (stepped HCD, NCE 30 +/− 25%) and an isolation width of 1.2 Da. Unassigned, singly charged as well as ≥8 charge states were excluded from fragmentation and fragmented precursor masses were excluded dynamically for 30 s. Fragment spectra were recorded at a resolution of 35,000 for a target AGC value of 1 × 10^6^ and a maximal injection time of 120 ms.

#### 2.2.3. Database Search and TMT Quantification

MS raw data files from all 18 SCX fractions (including re-injection/exclusion list runs) for each TMT set were merged for relative quantification and identification using Proteome Discoverer version 1.3 (Thermo Fisher Scientific, Inc., Waltham, MA, USA). A database search for each set was performed with the Mascot search engine version 2.3 (Matrix Science LTD., London, UK) using the UniProtKB/Trembl database (downloaded 2012-11-19, Swiss Institute of Bioinformatics, Lausanne, Switzerland) with MS peptide tolerance of 10 ppm and MS/MS tolerance of 100 millimass units (mmu). Tryptic peptides were accepted with one missed cleavage and variable modifications of methionine oxidation, cysteine methylthiolation and fixed modifications of N-terminal TMT6plex and lysine TMT6plex were selected.

The detected peptide threshold in the software was set to 1% False Discovery Rate by searching against a reversed database and the corresponding result was filtered by selecting proteins with at least one unique peptide per protein. Identified proteins were grouped by sharing the same sequences to minimize redundancy. For TMT quantification, the ratios of the TMT reporter ion intensities in MS/MS spectra ([M + H]^+^ *m*/*z* 126–131) from raw data sets were used to calculate fold changes between samples. The ratios were derived by Proteome Discoverer version 1.3 using the following criteria: fragment ion tolerance as 100 mmu for the most confident centroid peak, TMT reagent purity corrections factors were used and missing values were replaced with minimum intensity. Only peptides unique for a given protein were considered for relative quantitation, excluding those common to other isoforms or proteins of the same family. The quantification was normalized using the protein median.

#### 2.2.4. Statistical Analysis

The results were exported into MS Excel (Microsoft, Redmond, WA, USA) for manual data interpretation and statistical analysis. Welch’s unequal variances *t*-tests were performed after log-transformation of the data.

### 2.3. Validation Study

#### 2.3.1. ELISA

Plasma bleomycin hydrolase (BH) levels were determined blindly using an ELISA kit (Cusabio Biotech Co., Ltd., Wuhan, China), catalogue number CSB-EL002716HU, according to the manufacturer’s instructions.

#### 2.3.2. Statistical Analysis

All analyses were carried out using SPSS^®^ statistical software 16.0 for Windows^TM^ (SPSS, Chicago, IL, USA). Median (interquartile range) values were calculated for continuous variables and categorical data was expressed as absolute numbers with percentages. Differences in findings between study groups were assessed by Chi-square tests (two-tailed without Yates correction) for categorical variables and by Mann-Whitney tests for continuous variables. Correlation between plasma BH level and the maximum diameter of the AAA was assessed by Spearman’s method, while the Wilcoxon signed rank test was used to compare BH levels before and after surgery.

## 3. Results

### 3.1. Discovery Study

The AAA patients had infrarenal aneurysms (both small and large) with an average diameter (range) of 5.5 (3.5–9.6) cm. The controls had normal infrarenal aortic diameters (defined as <3.0 cm). In total, 522 proteins were detected in the plasma samples (Supplementary Table S2). To pin-point potential biomarkers among these, two criteria were used for selection: (i) at least an average 1.5-fold change in protein abundance between patients with AAA and controls; and (ii) this up- or down-regulation also had to be statistically significant (*p* < 0.05) according to the Welch test. Eight of the 522 plasma proteins detected fulfilled both these criteria. Five of these proteins were up-regulated and three proteins were down-regulated in AAA patients compared to the controls. The identification of these eight proteins is summarized in Table 2.

### 3.2. Validation Study

The AAA patients were further divided into patients with ruptured and non-ruptured AAAs, and the latter also sub-divided into patients with small and large aneurysms. The levels of hemoglobin and BH in the different groups are shown in Table 1. Lower levels of both these proteins were observed in the group with ruptured AAA compared to both the control group and the group with non-ruptured AAA. Furthermore, the BH levels in the patient group with large AAA were found to be lower compared to the control group. There was also a negative correlation (ρ= −0.366, *p* = 0.001) between the BH level and the maximum aneurysm diameter assessed in the group of patients with non-ruptured AAA.

**Figure 1:**
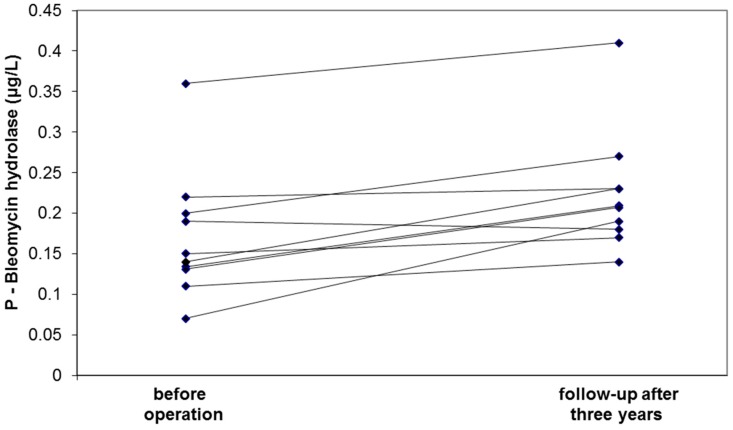
Circulating levels of bleomycin hydrolase before and three years after intervention of patients with AAA.

Nine of the ten follow-up patients showed elevated BH concentrations three years after surgery (Figure 1). This increase from 0.14 (0.13–0.20) μg/L preoperatively to 0.21 (0.18–0.24) μg/L postoperatively was also found to be statistically significant (*p* = 0.008). The magnitude of this increase in BH concentration is in line with the difference observed between the group with large AAAs and the control group (Table 1).

## 4. Discussion

This study identified differences in protein profiles among 522 different plasma proteins from patients with AAA and controls without AAA using a proteomic approach based on TMT labelling and LC-MS/MS for protein identification and relative quantification. Eight proteins were found to differ significantly in regulation between patients with AAA and matched controls without AAA. Among these eight proteins, five were up-regulated and three were down-regulated in patients with AAA compared to the matched controls without AAA.

One of the up-regulated proteins in AAA patients was hemoglobin subunit β. Hemoglobin is the major oxygen-carrying protein in red blood cells (RBCs). The up-regulation of hemoglobin subunits in plasma is a consequence of hemolysis of RBCs and cannot be considered as a plausible AAA biomarker, although oxidative stress may be induced by the presence of free hemoglobin. Oxidative stress is involved in the chronic pathological vascular remodeling of both AAA and occlusive atherosclerosis [16,17]. One of the most convincing findings in the present study was the up-regulation of C-reactive protein (CRP) in patients with AAA compared to controls. This finding was expected since CRP is an inflammatory marker, and several earlier studies have demonstrated an activated inflammatory response in patients with non-ruptured infrarenal aortic aneurysm expressed by elevated levels of CRP and Interleukin-6 [4,5,18]. The up-regulation of complement component C9 [19], leukocyte immunoglobulin-like receptor subfamily A member 2 [20], and fibrinogen γ chains [5,21] are also attributed to the inflammatory response in humans. These inflammatory markers, related to AAA, have obvious limitations as they are not disease specific, for example having an established correlation with atherosclerosis [6].

None of the three down-regulated proteins registered in the AAA patients have previously been considered to be associated with AAA. One of these down-regulated proteins was keratin 77. Keratins are the largest subgroup of intermediate filament proteins, which are important constituents of the cellular cytoskeleton. Current knowledge has identified the emerging importance of keratin intermediate filaments in normal and diseased epithelia, but also as a potential marker of various diseases [22]. Another down-regulated protein was serpin B4, a protein with less understood biological function. However, serpin B4 appears to play an important role in aberrant epithelial proliferation [23]. The regulation of biological events by enzymatic activity is a common paradigm in both normal and pathological states. From this point of view, the most interesting of the identified down-regulated proteins is the enzyme BH. This enzyme was first discovered in mouse liver due to its ability to inactivate the anti-tumor antibiotic bleomycin [24]. However, BH is widely expressed in human tissue and mainly exhibits broad-specificity aminopeptidase activity. Moreover, BH seems to have a protective role against homocysteine toxicity [25]. In a recent study, hyperhomocysteinemia was associated with the presence of AAA and also showed a positive dose–response relationship between homocysteine and AAA size [26]. From this point of view, a low level of BH in patients with AAA is a possible pathophysiological clue and a potential plasma biomarker.

The down-regulation of the enzyme BH was verified by ELISA in the expanded validation study. It is well-known that the concentration of blood cells and plasma proteins are decreased by massive bleeding due to hemodilution, and the decreased levels of hemoglobin and BH in patients with ruptured compared to non-ruptured AAAs is likely a reflection of this dilution. Although clearly significant, the observed correlation between BH and aneurysm diameter was fairly weak and the validation thus failed to demonstrate a clinically useful relationship between BH levels and AAA diameter. The follow-up study, however, showed significant elevation (towards normalized values) of the BH concentration three years postoperatively—a finding that strengthens the hypothesis that BH has a pathophysiological role in AAA.

## 5. Conclusions

In summary, the present proteomic study identified up-regulation of several inflammatory markers in AAA patients, which is a well-known feature of AAA [4,5]. Furthermore, three down-regulated proteins with unknown roles in AAA were identified. The most interesting of these potential biomarkers is the enzyme BH, which was down-regulated in patients with AAA compared to the controls without aneurysm. The findings warrant urgent new investigations to clarify the pathophysiological role of BH in AAA.

## Figures and Tables

**Table 1:** Demographics and laboratory results for controls and patients with abdominal aortic aneurysm (AAA) in the validation study.

Parameter	Controls (*n* = 41)	Non-Ruptured AAA (*n* = 78)		Ruptured AAA	*p*-Values (Non-Ruptured vs. Ruptured AAA)
		Small AAA (*n* = 38)	Large AAA (*n* = 40)	(*n* = 55)	
Age, years	72 (67–79)	70 (66–76) ^NS^	71 (63–78) ^NS^	73 (70–79) ^NS^	0.509
Sex, male	33 (80%)	27 (71%) ^NS^	35 (88%) ^NS^	44 (80%) ^NS^	0.942
Current smoking	18 (44%)	16 (42%) ^NS^	19 (48%) ^NS^	22 (40%) ^NS^	0.576
Aneurysm diameter, cm	No aneurysm	4.0 (3.5–4.3)	6.0 (5.2–7.1)	7.3 (6.0–8.0)	<0.001
Hemoglobin, g/L	141 (130–147)	142 (134–151) ^NS^	139 (131–145) ^NS^	118 (101–136) **	<0.001
Bleomycin hydrolase, μg/L	0.19 (0.16–0.25)	0.20 (0.16–0.24) ^NS^	0.14 (0.09–0.19) *	0.12 (0.08–0.19) **	0.001

The figures indicate median (interquartile range) or the number (percentage) of patients/controls. ^NS^ Non-significant (*p* > 0.05), * *p* < 0.01, ** *p* < 0.001 when compared to the control group.

**Table 2:** Identification of potential biomarker proteins in the discovery study.

Protein	UniProt Accession	Fold Change (AAA/C)	Welch Test *p*-Value
Complement component C9	P02748	1.506	0.001
Keratin 77	Q0IIN1	0.440	0.004
Bleomycin hydrolase	Q13867	0.565	0.008
Hemoglobin subunit β	P68871	1.974	0.016
Fibrinogen γ chain	P02679	1.558	0.017
Leukocyte immunoglobulin-like receptor subfamily A member 2	A8MZH0	2.496	0.021
C-reactive protein	P02741	2.485	0.026
Serpin B4	F8W9L1	0.366	0.049

## Supplementary Materials

The following are available online at http://www.mdpi.com/2227-7382/6/4/43/s1, Table S1: TMT-labeling setup. Table S2: All 522 detected proteins by nano LC-MS analysis.
