# Supplementary material for: Identification of Potential Plasma Biomarkers for Abdominal Aortic Aneurysm Using Tandem Mass Tag Quantitative Proteomics
Source: Proteomes. 2018 Oct 18;6(4):43. doi: 10.3390/proteomes6040043 (PMC6313917; doi:10.3390/proteomes6040043)
Supplement: Supplementary file 1 [file proteomes-06-00043-s001.zip › Table S1.pdf]

Supplementary Table S1. TMT-labeling setup

TMT 6 plex kit, Pierce (Thermo Scientific), Lot no: NE172480A

Set 1 (A4 in result files)

**Sample**	P1	C1	S1	L1	S5	C5
**TMT label**	126	127	128	129	130	131

Set 2 (B4 in result files)

**Sample**	P2	C2	S2	L2	L5	C6
**TMT label**	126	127	128	129	130	131

Set 3 (C4 in result files)

**Sample**	P3	C3	S3	L3	S6	C7
**TMT label**	126	127	128	129	130	131

Set 4 (D4 in result files)

**Sample**	P4	C4	S4	L4	L6	C8
**TMT label**	126	127	128	129	130	131

Sample coding: P=Pooled sample, C=Control (no aneurysm), S=Small aneurysm, L=Large aneurysm
